# Substituent Effects Impact Surface Charge and Aggregation of Thiophenol-Labeled Gold Nanoparticles for SERS Biosensors

**DOI:** 10.3390/bios12010025

**Published:** 2022-01-05

**Authors:** Nolan File, Joseph Carmicheal, Alexey V. Krasnoslobodtsev, Nicole C. Japp, Joshua J. Souchek, Sudesna Chakravarty, Michael A. Hollingsworth, Aaron A. Sasson, Gopalakrishnan Natarajan, Prakash G. Kshirsagar, Maneesh Jain, Chihiro Hayashi, Wade M. Junker, Sukhwinder Kaur, Surinder K. Batra

**Affiliations:** 1 Sanguine Diagnostics and Therapeutics Inc., Omaha, NE 68106, USA; nolanfile@gmail.com (N.F.); njapp@sdtne.com (N.C.J.); jsouchek@sdtne.com (J.J.S.); mahollin@unmc.edu (M.A.H.); Aaron.Sasson@stonybrookmedicine.edu (A.A.S.); wjunker@sdtne.com (W.M.J.); 2 School of Chemistry, University of Edinburgh, Edinburgh EH8 9YL, UK; 3 Department of Biochemistry and Molecular Biology, University of Nebraska Medical Center, Omaha, NE 68198, USA; joseph.carmicheal@unmc.edu (J.C.); sudesna.chakravarty@unmc.edu (S.C.); g.natarajan@unmc.edu (G.N.); prakash.kshirsagar@unmc.edu (P.G.K.); mjain@unmc.edu (M.J.); chihiro.hayashi@unmc.edu (C.H.); 4 Department of Physics, University of Nebraska at Omaha, Omaha, NE 68182, USA; akrasnos@unomaha.edu; 5 Department of Pathology and Microbiology, University of Nebraska Medical Center, Omaha, NE 68198, USA; 6 Fred and Pamela Buffett Cancer Center, University of Nebraska Medical Center, Omaha, NE 68198, USA; 7 Eppley Institute for Research in Cancer and Allied Diseases, University of Nebraska Medical Center, Omaha, NE 68198, USA; 8 Department of Surgery, Renaissance School of Medicine, Stony Brook University, Stony Brook, NY 11794, USA

**Keywords:** SERS, immunoassay, nanoparticles, multiplexing, Raman reporter molecules, aggregation

## Abstract

SERS immunoassay biosensors hold immense potential for clinical diagnostics due to their high sensitivity and growing interest in multi-marker panels. However, their development has been hindered by difficulties in designing compatible extrinsic Raman labels. Prior studies have largely focused on spectroscopic characteristics in selecting Raman reporter molecules (RRMs) for multiplexing since the presence of well-differentiated spectra is essential for simultaneous detection. However, these candidates often induce aggregation of the gold nanoparticles used as SERS nanotags despite their similarity to other effective RRMs. Thus, an improved understanding of factors affecting the aggregation of RRM-coated gold nanoparticles is needed. Substituent electronic effects on particle stability were investigated using various para-substituted thiophenols. The inductive and resonant effects of functional group modifications were strongly correlated with nanoparticle surface charge and hence their stability. Treatment with thiophenols diminished the negative surface charge of citrate-stabilized gold nanoparticles, but electron-withdrawing substituents limited the magnitude of this diminishment. It is proposed that this phenomenon arises by affecting the interplay of competing sulfur binding modes. This has wide-reaching implications for the design of biosensors using thiol-modified gold surfaces. A proof-of-concept multiplexed SERS biosensor was designed according to these findings using the two thiophenol compounds with the most electron-withdrawing substitutions: NO_2_ and CN.

## 1. Introduction

Raman spectroscopy measures the change in the energy, known as Raman shift, of inelastically scattered photons after they interact with a molecule [1]. These shifts correspond to the activation of vibrational modes, thereby producing a spectral pattern characteristic of the chemical groups present in the interacting molecule. Hence, structural information of the species can be obtained based on its unique vibrational signature. The application of Raman spectroscopy in sensing platforms is chiefly limited by the efficiency of Raman scattering; only one in a million incident photons are scattered inelastically. In surface-enhanced Raman spectroscopy (SERS), proximity to a noble metal surface is used to amplify inelastic scattering by several orders of magnitude [2]. As such, SERS has gained popularity as a technique, which overcomes the factors limiting the efficiency of Raman scattering. Its application has enabled the development of certain innovative techniques, including SERS-based biosensors.

While Raman spectra of biological species are often complicated and susceptible to interference by other analyte components, label-free characterization has been possible [3,4,5]. For instance, cancer detection using principal component discriminant function analysis of SERS spectra obtained from exosomes has been demonstrated [6]. However, the need to use computational techniques to overcome spectral interference and readout ambiguity of label-free SERS has prompted the investigation of more specific techniques for SERS of biological samples. These include a myriad of immunoassay-based biosensors involving the detection of protein biomarkers in patient serum using surface-functionalized gold nanoparticles (AuNPs) [7].

**Figure 1:**
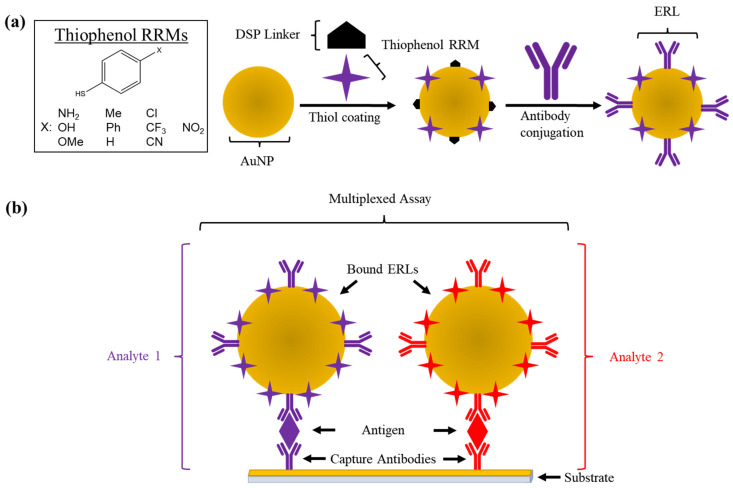
Preparation of ERLs and schematic of a multiplex SERS immunoassay biosensor. (**a**) The surface of an AuNP is decorated with a mixture of thiophenol RRM and detection antibodies. (**b**) Different ERL configurations in a multiplexed sensor allow quantification of additional markers using unique antibody/RRM pairs.

In such sensors, so-called extrinsic Raman labels (ERLs), gold nanoparticles functionalized with monoclonal antibodies and a thiol Raman reporter molecule (RRM, Figure 1a), are used as a detection mechanism in place of the colored substrates or radioisotopes employed in more traditional immunoassays (i.e., ELISA and radioimmunoassay). The combination of highly localized Raman signal amplification, reporter molecule diversity, high sensitivity, and small sample volume makes SERS-based assays an attractive alternative to their traditional counterparts. As such, SERS immunoassays have become a promising technique for low-level, small volume biomarker detection [8].

At present, single-marker SERS biosensors have been demonstrated, such as for the detection of serum MUC4 mucin for pancreatic cancer screening in a sandwich assay format [9]. However, one attractive application of SERS is the ability to detect and quantify multiple biomarkers at once [10,11]. By employing multiple ERL configurations (with unique pairs of antibodies and RRMs), the simultaneous measurement of multiple biomarkers on a single sensor is possible (Figure 1b). Given the growing research interest in multiple biomarker panels for the detection of cancer, the development and improvement of multiplexed SERS biosensors could mark a significant advancement in the field of a cancer diagnosis.

Prior development efforts on multiplexing have largely focused on spectroscopic considerations; since the SERS spectrum obtained from a multiplexed sensor would consist of overlapped spectra of multiple RRMs, the availability of strong, reproducible, and well-separated SERS bands is paramount [12,13]. However, RRMs selected by strictly spectral criteria may result in a major procedural difficulty: uncontrolled aggregation and/or precipitation of the nanoparticle colloid after surface decoration [14,15]. Since aggregation results in significant SERS signal variation and non-specific adsorption, the ERL colloid cannot be reliably used [7,16,17]. Furthermore, precipitated nanoparticle aggregates cannot be resuspended, effectively resulting in a failed experiment.

This phenomenon has hampered the successful development of multiplexed SERS biosensors since the understanding of the factors that result in aggregation with certain RRM candidates but not other structurally similar RRMs has been heretofore limited. As a result, multiplexing development has relied upon trial-and-error testing of RRM combinations. The success of single-plex SERS assays using 4-nitrobenzenethiol (NBT) has led to the proposed use of additional thiophenols, such as 3- and 4-methoxythiophenol as RRMs [8].

The thiophenol moiety can be easily modified with various chemical groups. This allows an RRM panel for multiplexing to be easily designed based solely on the SERS signature of the various functional groups. However, aggregation is frequently observed when attempting to prepare ERLs with such candidates despite their similarity to NBT. As such, an improved understanding of ERL colloid stability is needed. Herein it is proposed that the inductive and resonant effects of functional group modifications felt by the gold-binding thiol group alter the surface charge of the AuNPs, thereby affecting the tendency of the ERL colloid to aggregate. This concept was demonstrated experimentally and was shown to be an effective design strategy in a proof-of-concept multiplex SERS biosensor.

## 2. Materials and Methods

### 2.1. AuNP RRM Coating

60 nm citrate-stabilized gold nanoparticles were purchased from BBI Solutions (2.6 × 10^10^ particles mL^−1^). 50 mM borate buffer packs (pH 8.5) were purchased from Thermo Scientific. 1 mL aliquots of the AuNPs were buffered with borate (2 mM) before being treated with one of a selection of para-substituted thiophenol analogs purchased from Sigma Aldrich (2 µL of 2 mM, dissolved in acetonitrile). The following para-substitutions were assessed in this study: NH_2_, OH, OMe, CH_3_, Ph, H, Cl, CF_3_, CN, and NO_2_ (Table 1). This mixture was vortexed and then mixed on a rotator for 5 min at room temperature. The nanoparticles were pelleted via centrifugation at 5500 RPM for 10 min. The supernatant was removed, and the nanoparticle pellet was resuspended in 1 mL of 2 mM borate buffer. Experiments were conducted immediately after the thiol coating procedure, and samples were made fresh for each experiment.

### 2.2. AuNP Dynamic Light Scattering Analysis (Zeta-Potential)

A Malvern Nano series Zetasizer, Model Nano-ZS90 was used for surface zeta potential assessment. The AuNPs or AuNP/thiol mixtures were loaded into a DTS1070 capillary cell. Sample measurements were conducted at 25 °C with 30 zeta runs per measurement. Three measurements were conducted for each sample.

### 2.3. UV-Visible Spectroscopy Analysis

UV-visible spectra were utilized as a readout of particle precipitation [19]. Absorbance readings of the bare and RRM-coated AuNPs at 539 nm were obtained using a Thermo Scientific™ NanoDrop™ One spectrophotometer.

### 2.4. Nanoparticle Tracking Analysis

Nanoparticle tracking analysis (NTA) was conducted using a NanoSight LM10 Nanoparticle Analysis System equipped with NTA 2.3 Analytical Software. NTA provided particle quantification and size distribution of the colloidal solution. After RRM coating, the solution was diluted in 2 mM borate buffer solution and immediately injected into the sample chamber for analysis.

### 2.5. Proof-of-Concept Multiplexed SERS Assay

The SERS assay construction and measurement procedures were modified from our previous studies [9]. Briefly, a sandwich-based SERS assay was constructed by first defining hydrophobic addresses on a gold-coated microscope slide by stamping with 1-octadecanethiol solution (2 mM, EtOH). Each address was treated with the linker dithiobis(succinimidyl propionate) (DSP) followed by capture antibody (8G7 and 1116-NS-19-9) deposition and blocking with bovine serum albumin (1%). Samples were prepared from a lysate of CFPAC-1, a pancreatic cancer cell line with known expression of our target antigens (MUC4 and CA19-9), diluted to 80, 40, and 20 μg mL^−1^ of total protein content. 5 μL of the sample were applied to each address and incubated overnight at 4 °C. ERL synthesis was conducted by thiol adsorption (RRM + DSP) followed by detection antibody (8G7 and 1116-NS-19-9, 1 ng mL^−1^) decoration. Slides were washed to remove sample, and ERLs (5 μL) were deposited onto each address. After a 3-h incubation at 4 °C, the slides were again washed before being dried using compressed nitrogen. Raman spectra were collected utilizing a custom-built Raman reader consisting of an Olympus BX-40 microscope frame equipped with Thorlabs automatic piezo stage. The laser (647 nm, 1 mW) was focused to a ~1 μm diameter spot size by the microscope objective (40×) with a numerical aperture of 0.75 (Olympus PLAN C N 40×/0.65). Five accumulated SERS measurements were recorded with a 5 s acquisition time each.

## 3. Results

### 3.1. RRM Effect on AuNP Surface Charge

The AuNP colloid, an integral part of the SERS nanotag, is susceptible to aggregation. However, electrostatic repulsion generates a potential energy barrier against aggregation. For this reason, capping agents such as citrate, hexadecyltrimethylammonium bromide, and polyvinylpyrrolidone are often used. These species coordinate to the gold surface and impart a surface charge. Functionalization of AuNPs with higher affinity surface ligands such as thiols results in a displacement of these capping agents, thereby influencing their surface charge. As such, the impact of RRM decoration on particle charge and susceptibility to aggregation was investigated.

The conditions for the RRM coating procedure were selected based on previously reported protocols for the preparation of ERLs for SERS immunoassays [9]. For instance, 60 nm AuNPs were used due to their optimal enhancement of thiophenol RRMs [20]. Hence, the results obtained should accurately reflect the stability of the thiol-functionalized AuNPs for that application. Further optimization of these conditions may be possible with respect to nanoparticle stability. However, it would be necessary to assess the effects of these changes on the biological components of the assay which lies outside the scope of this work.

To evaluate the effects of functional group modifications on particle stability, AuNPs were treated with a selection of para-substituted thiophenol compounds. These compounds were chosen due to their structural similarity to NBT. Since the electronic effects of aromatic substituents differ at ortho, meta, and para positions, it is important to use thiophenol compounds which are substituted at the same position. Additionally, the para substituent is least likely to influence the thiol group in other ways, such as by sterically interfering with its ability to bind the gold surface.

**Figure 2:**
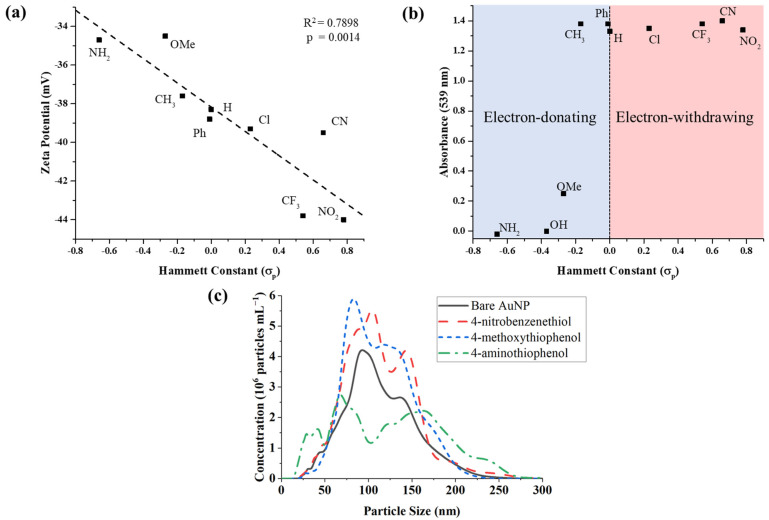
Measured effects of thiophenol functional group modifications on gold nanoparticles. (**a**) Zeta-potential measurements of AuNPs treated with para-substituted thiophenols. Hammett constants (σ_p_) of the varied substituent are negatively correlated with AuNP surface charge (R^2^ = 0.79, *p* = 0.0014). (**b**) UV-visible absorbance data show the impact of thiophenol inductive effects on AuNP precipitation. P-thiophenols with σ_p_ ≥ −0.25 did not cause aggregation, while those with σ_p_ < −0.25 did. (**c**) Size distributions of thiol-coated AuNPs obtained via nanoparticle tracking analysis (NTA).

Herein, zeta potential measurements were used to characterize the charge-stability of AuNPs treated with the para-thiophenols. The approximate electronic effects of the functional group modifications were quantified using Hammett constants (σ_p_) (Table 1). Though derived from the properties of benzoic acid derivatives, Hammett constants nonetheless provide a reasonable, ad hoc standard for the inductive and resonant effects of the varied substituent. The Hammett constant of the varied moiety was found to be negatively correlated with the surface charge of the functionalized AuNPs (R^2^ = 0.79, *p* = 0.0014, Figure 2a). Functional groups with a more positive σ_p_ (i.e., electron-withdrawing groups (EWGs)), e.g., −NO_2_ and −CN, produced colloids with more strongly negative zeta potential while those with more negative σ_p_ (i.e., electron-donating groups (EDGs)), e.g., -NH_2_, had weaker negative zeta potential. In all cases, the surface charge of the thiolated nanoparticles was less negative than the untreated gold nanoparticles. However, those with EWGs exhibited a lower magnitude of surface charge diminishment than those with EDGs. Hence, thiophenols derivatives bearing EWGs produced ERLs that were more stable to aggregation.

### 3.2. RRM Impact on AuNP Aggregation

The aggregation of AuNPs results in an alteration of their optical properties due to distance-dependent plasmon coupling [21]. With sufficient aggregation, particles are observed to precipitate yielding a colorless solution and black solid. These phenomena can be assessed by UV-vis spectroscopy, which has been used as an effective measure of particle aggregation in previous studies [19]. The localized surface plasmon resonance (LSPR) peak wavelength for the 60 nm AuNPs was identified as 539 nm (Figure S1). Absorption at this wavelength was therefore used to characterize the extent of aggregation that occurred after the addition of the various RRMs.

It was observed that para-substituted thiophenols with electron-withdrawing or weakly electron-donating functional groups (σ_p_ ≥ −0.15) did not affect the LSPR peak absorption (Figure 2b). This suggested that no aggregation occurred with the addition of these RRMs. 4-methoxythiophenol, which has a moderately electron-donating substituent (σ_p_ = −0.27), experienced partial precipitation after centrifugation of the thiol-coated nanoparticles, resulting in a decrease of the LSPR peak absorbance from 1.38 to 0.25 (Figure 2b). The two thiophenols bearing substituents with the greatest electron-donating potential, 4-aminothiophenol (σ_p_ = −0.66) and 4-mercaptophenol (σ_p_ = −0.37), induced rapid aggregation and precipitation upon addition to the nanoparticle colloid, resulting in complete loss of the LSPR spectrum. As such, we were unable to acquire a spectrum of the aggregated nanoparticles before precipitation occurred. However, a red-to-violet color change was observed, indicative of the spectral shift associated with nanoparticle aggregation. Images of the visual appearance of AuNPs treated with NBT, 4-methoxythiophenol, or 4-aminothiophenol are provided in Figure S1b.

To further corroborate the LSPR findings, NTA was conducted for assessment of colloidal aggregation. The quantity of smaller particles was dramatically decreased upon addition of 4-aminothiophenol as compared to AuNPs alone or with NBT (Figure 2c). Notably, 4-aminothiophenol shifted a proportion of the total particle distribution to the right (towards larger size), generating a bimodal peak with heavy right-tailed skewness. However, conclusions from this data are limited due to the tendency of aggregated particles to precipitate rapidly. Hence, larger aggregates may not be detected since they did not remain suspended.

### 3.3. Proof-of-Concept SERS Multiplexing

**Figure 3:**
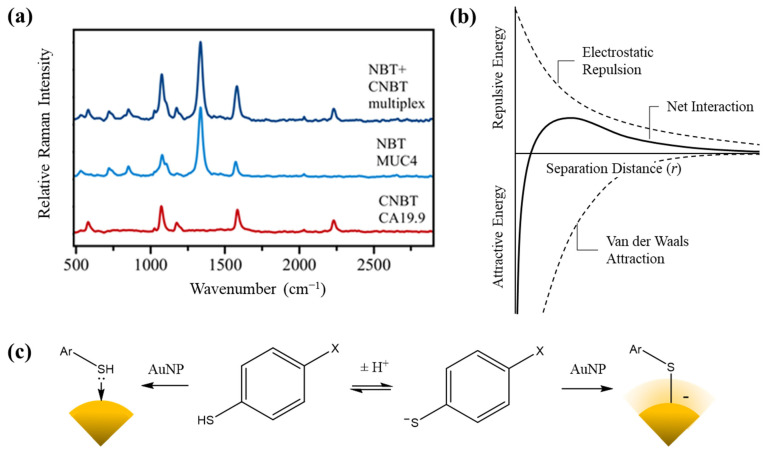
Multiplexed SERS spectra and schematics for interpretation of observed phenomena. (**a**) Raman spectra obtained from a sandwich SERS immunoassay for MUC4 and CA19-9 using CFPAC-1 cell line lysate. The spectra have been vertically shifted for clarity. Raman band assignment for NBT: 540 cm^−1^ (benzene ring deformation), 724 cm^−1^ (ρ-NO_2_, rocking), 857 cm^−1^ (ν-NO_2_, scissoring), 1079 cm^−1^ (ν-CS, stretching), 1112 cm^−1^ (δ-CH, in-plane bending), 1136 cm^−1^ (ν-NO_2_, symmetric stretching), 1574 cm^−1^ (ν-CC, stretching). Raman band assignment for CNBT: 577 cm^−1^ (benzene ring deformation), 1069 cm^−1^ (ν-CS, stretching), 1172 cm^−1^ (δ-CH, in-plane bending), 1580 cm^−1^ (ν-CC, stretching), 2225 cm^−1^ (ν-CN, stretching). (**b**) Qualitative representation of the potential energy diagram governing the interaction between colloidal particles described by DLVO theory. (**c**) A mechanistic model of the competing sulfur binding modes to gold surfaces. The central equilibrium determines the balance of thiol and thiolate species in solution. The binding of the neutral thiol results in no contribution of surface charge (left) while the thiolate confers negative charge (right).

To demonstrate that the choice of RRMs predicated on electron-withdrawing capability could facilitate multiplexing, a SERS sandwich immunoassay experiment was conducted. The thiols with the greatest electron withdrawing potential in our analysis, NBT (σ_p_ = 0.78) and 4-cyanobenzenethiol (CNBT, σ_p_ = 0.66) were chosen as RRMs amenable to multiplexed SERS. Two separate ERLs were synthesized, one functionalized with NBT and 8G7 (anti-MUC4 mucin monoclonal antibody) and the other with CNBT and 1116-NS-19-9 (anti-CA19-9 monoclonal antibody). A SERS immunoassay was carried out with these ERLs using a lysate of the pancreatic cancer cell line CFPAC-1 which expresses both MUC4 and CA19-9 [22,23]. Separate plates were treated with one of the two ERLs or a 1:1 mixture of both. SERS spectra were then obtained (Figure 3a).

One beneficial feature of SERS is the presence of narrow Raman bands, which allows signals from different RRMs to be distinguished. NBT and CNBT have well-separated bands corresponding to vibration modes of their para-substituent groups: ν = 1336 cm^−1^ (−NO_2_ symmetric stretch) and ν = 2225 cm^−1^ (−CN stretch). These characteristic peaks were both detected in the Raman spectra taken from the multiplex assay (treated with the ERL mixture). Comparison of the Raman intensities between the single plex assays with the multiplexed one suggests no interference as the intensity remains the same. Furthermore, the intensity of the peaks was observed to be antigen concentration-dependent, providing confidence that a full-scale multiplex assay can be built with the use of these two RRMs.

## 4. Discussion

In general, colloids are considered thermodynamically unstable; their persistence is due to the kinetic barrier to aggregation. Particles with a surface charge attract ions of opposite charge, forming a so-called electric double layer. As neighboring particles approach one another, their double layers coulombically repel each other. The balance between this repulsive force and the attractive van der Waals interactions between suspended particles is described quantitatively by DLVO theory, giving Equation (1) for the potential energy (U):

(1) $$\beta U\left(r\right)=Z^{2}\lambda_{B}{\left(\frac{e^{\kappa a}}{1+\kappa a}\right)}^{2}\frac{e^{-\kappa r}}{r}$$

where $\beta =k_{B}T$, Z is the elementary charge of the interacting particles, $\lambda_{B}$ is the Bjerrum length, $\kappa^{-1}$ is the Debye length, and r is the separation distance [24,25]. Plotting this equation against r yields a potential energy diagram as a function of interparticle separation (Figure 3b).

The energy minimum observed at low separation corresponds to the process of van der Waal-driven aggregation. However, for this to occur, the energy maximum produced by double-layer repulsion must be overcome. This acts as the energetic barrier for the aggregation process; the kinetic energy of interacting particles must be sufficient to overcome this barrier in order to induce aggregation. Hence, colloid stability at a given temperature is dependent on the height of the potential energy maximum, which is in turn dependent on the magnitude of the surface charge. This is largely due to interactions at the nanoparticle surface rather than the oxidation state of the gold itself. As such, modifications such as thiol self-assembled monolayers (SAMs) may directly affect the surface charge. Indeed, while thiolation has been reported to result in the oxidation of surface atoms to Au(I), a negative surface charge is observed for thiolated AuNPs [26].

The exact binding mode of thiols to gold surfaces has been subject to extensive debate. The conventional understanding of gold/sulfur binding involves the formation of a covalent interaction between the deprotonated thiolate and the gold surface, which may be referred to as chemisorption. However, recent investigations involving single-molecule conductance measurements suggest that thiols may also bind to gold surfaces as the free thiol via a dative interaction, constituting physisorption rather than chemisorption [27]. This has direct implications for the stability of gold nanoparticles functionalized with thiol SAMs since the binding of a thiolate imparts a negative charge on the gold surface while binding of a neutral thiol does not.

We propose, therefore, that the relationship between zeta potential (ζ) and the Hammett constant (σ_p_) of the varied p-thiophenol substituent observed herein arises due to changes in the relative population of the thiol and thiolate binding modes. While the precise mechanism of thiol monolayer assembly remains unclear, this finding suggests that the resultant binding mode upon incidence with a gold surface may depend on the thiol’s charge state in solution. This explains the observed correlation since electronic effects alter the acidity of weak acids such as thiols by stabilizing/destabilizing the negative charge of their conjugate base. Hence, modification of thiophenol with EWGs lowers the pK_a_ of its thiol group, increasing the fraction ionized in solution and favoring chemisorption (Figure 3c).

Since the AuNPs used in these experiments are citrate-stabilized, a negative surface charge is present in the untreated particles (−50.8 mV). This weakly associated citrate layer is substituted by the thiol SAMs upon treatment with a thiophenol analogue. Hence, occurrence of the thiol binding mode results in lost surface charge as negative citrate ions are displaced by the neutral species. Conversely, the replacement of citrate with thiolate preserves the negative surface charge. This is supported by the zeta potential measurements since the untreated AuNPs had a greater negative potential than any of the thiolated particles and those treated with thiophenols bearing electron-withdrawing substituents faced the least reduction in net negative surface charge.

As mentioned previously, the interpretation of the NTA data was limited by the confounding factor of particle precipitation. Accordingly, further assessment of particle aggregation may be desired using other nanoparticle characterization techniques. Dynamic light scattering (DLS) and transmission electron microscopy (TEM) were considered for this study but were deemed unsuitable. DLS produces similar size characterizations to NTA and would likely suffer the same limitations due to precipitation. While TEM would allow such aggregates to be visualized, the process of drying AuNP samples onto the TEM grids can itself cause aggregation. As such, the visual appearance of the colloid remains the simplest and best method for determining whether aggregation has occurred in this case.

Some limitations of the present study may be acknowledged. The first is that while discrepancies in surface charge imply variations in the charge state of the surface modifications, it does not conclusively prove the fate of the thiol hydrogen. Further study of the sulfur-gold interface, such as by ab initio calculations, is needed to fully understand the nature of thiol SAMs on gold. Second, functional groups can have alternative effects on nanoparticle stability, including entropic, kinetic, and steric. To this end, the p-thiophenols used are relatively small molecules while the 60 nm particle size is rather large, thereby minimizing size effects due to thiol treatment. Additionally, the para-substituent is unlikely to sterically affect the thiol group’s ability to coordinate the gold surface. However, the presence of other ionizable groups such as in 4-aminothiophenol will influence surface charge as well. As such, investigation into these possible functional group impacts is warranted.

Additionally, it is noted that this study was primarily focused on understanding the role of functional group modifications on the stability of RRM-coated AuNPs. Rigorous characterization of the proof-of-concept multiplex biosensor’s analytical performance is an area for future study. Its purpose herein is solely to confirm that compatible RRMs can be selected based on the presence of EWGs.

## 5. Conclusions

Overall, this study shows that the inductive and resonant effects of functional group modifications in thiophenols contribute to the stability of the gold colloids they functionalize. A correlation between these electron-withdrawing or donating effects as approximated by Hammett constants and the surface charge of thiolated AuNPs has been demonstrated. Alteration in surface charge resulting from RRM decoration can lead to a decrease in electrostatic repulsion, thereby allowing attractive forces (i.e., Van der Waals) to predominate, leading to AuNP aggregation and subsequent precipitation. Stronger electrostatic repulsion in thiolated AuNPs can be achieved through modification with EWGs relative to the thiol position. This principle implicated CNBT as an RRM candidate for use in conjunction with NBT, which proved successful in our proof-of-concept multiplexed biosensor. The multiplexed SERS platform was able to detect each analyte individually (in separate samples with individual ERLs), as well as simultaneously (in the same sample with mixed ERLs), without loss of magnitude, spectral overlap, or particle aggregation. Hence, thiophenols bearing electron-withdrawing groups such as NBT and CNBT are effective RRMs. Therefore, the design of SERS-based biosensors should include consideration of the electronic effects of RRM functional group modifications.

These findings not only provide advancement for the design of thiol-functionalized AuNPs for applications such as multiplexed SERS, but they also shed light on the mechanism by which thiol-on-gold SAMs are formed. Since the inductive effects studied herein influence the acidity of weak acids such as thiols, it is suggested that these findings grant further support for the existence of competing for thiol and thiolate binding modes in sulfur-on-gold SAMs. This insight has wide-reaching implications for the field of biosensor development due to the multitude of designs that involve thiol-functionalized gold surfaces beyond the narrow scope of SERS immunoassays.

## Figures and Tables

**Table 1:** Thiophenol compounds used, their para substituents, and respective Hammett constants.

Reagent	Para Group	Hammett Constant (σ_p_) [18]
4-aminothiophenol	NH_2_	−0.66
4-mercaptophenol	OH	−0.37
5-methoxythiophenol	OMe	−0.27
4-methylbenzenethiol	CH_3_	−0.17
biphenyl-4-thiol	Ph	−0.01
thiophenol	H	0
4-chlorothiophenol	Cl	0.23
4-(trifluoromethyl) thiophenol	CF_3_	0.54
4-cyanobenzenethiol (CNBT)	CN	0.66
4-nitrobenzenethiol (NBT)	NO_2_	0.78

## Data Availability

Not applicable.

## Supplementary Materials

The following are available online at https://www.mdpi.com/article/10.3390/bios12010025/s1, Figure S1. Surface Plasmon Resonance (LSPR) peak for unmodified and modified 60 nm gold nanoparticles (AuNPs). (**a**) UV-visible spectrum of unmodified (DSP linker only, black), DSP linker and CNBT (red) modified, and DSP linker and NBT (blue) modified showing the LSPR peak at 539 nm. (**b**) Images of AuNPs treated with DSP linker and 4-nitrobenzenethiol (i), 4-methoxythiophenol (ii), and 4-aminothiophenol (iii) exhibiting no, partial, and total precipitation following centrifugation, re-spectively. AuNPs treated with 4-aminothiophenol prior to centrifugation (iv) exhibit rapid color change (spectral red-shift) associated with nanoparticle aggregation before precipitation occurs.
