# Supplementary material for: Substituent Effects Impact Surface Charge and Aggregation of Thiophenol-Labeled Gold Nanoparticles for SERS Biosensors
Source: Biosensors (Basel). 2022 Jan 5;12(1):25. doi: 10.3390/bios12010025 (PMC8773556; doi:10.3390/bios12010025)
Supplement: Supplementary file 1 [file biosensors-12-00025-s001.zip › biosensors-1502217-supplementary.pdf]

biosensors

Communication

Substituent Effects Impact Surface Charge and Aggregation of Thiophenol-Labeled Gold Nanoparticles for SERS Biosensors

Nolan File [1,2,†], Joseph Carmicheal [3,†], Alexey V. Krasnoslobodtsev [4], Nicole C. Japp [1], Joshua J. Souchek [1], Sudesna Chakravarty [3], Michael A. Hollingsworth [1,3,5,6,7], Aaron A. Sasson [1,8], Gopalakrishnan Natarajan [3], Prakash G. Kshirsagar [3], Maneesh Jain [3,6], Chihiro Hayashi [3], Wade M. Junker [1,3], Sukhwinder Kaur [3,*] and Surinder K. Batra [1,3,5,6,*]

1 Sanguine Diagnostics and Therapeutics Inc., Omaha, NE 68106, USA; nolanfile@gmail.com (N.F.); njapp@sdtne.com (N.C.J.); jsouchek@sdtne.com (J.J.S.); mahollin@unmc.edu (M.A.H.); Aaron.Sasson@stonybrookmedicine.edu (A.A.S.); wjunker@sdtne.com (W.M.J.)
2 School of Chemistry, University of Edinburgh, Edinburgh EH8 9YL, UK
3 Department of Biochemistry and Molecular Biology, University of Nebraska Medical Center, Omaha, NE 68198, USA; joseph.carmicheal@unmc.edu (J.C.); sudesna.chakravarty@unmc.edu (S.C.); g.natarajan@unmc.edu (G.N.); prakash.kshirsagar@unmc.edu (P.G.K.); mjain@unmc.edu (M.J.); chihiro.hayashi@unmc.edu (C.H.); wjunker@sdtne.com (W.M.J.)
4 Department of Physics, University of Nebraska at Omaha, Omaha, NE 68182, USA; akrasnos@unomaha.edu
5 Department of Pathology and Microbiology, University of Nebraska Medical Center, Omaha, NE 68198, USA
6 Fred and Pamela Buffett Cancer Center, University of Nebraska Medical Center, Omaha, NE 68198, USA
7 Eppley Institute for Research in Cancer and Allied Diseases, University of Nebraska Medical Center, Omaha, NE 68198, USA
8 Department of Surgery, Renaissance School of Medicine, Stony Brook University, Stony Brook, NY 11794, USA
* Correspondence: skaur@unmc.edu (S.K.); sbatra@unmc.edu (S.K.B.); Tel.: +1-402-559-3211. (S.K.); Tel.: +1-402-559-5455 (S.K.B.)
† These authors contributed equally to this work.

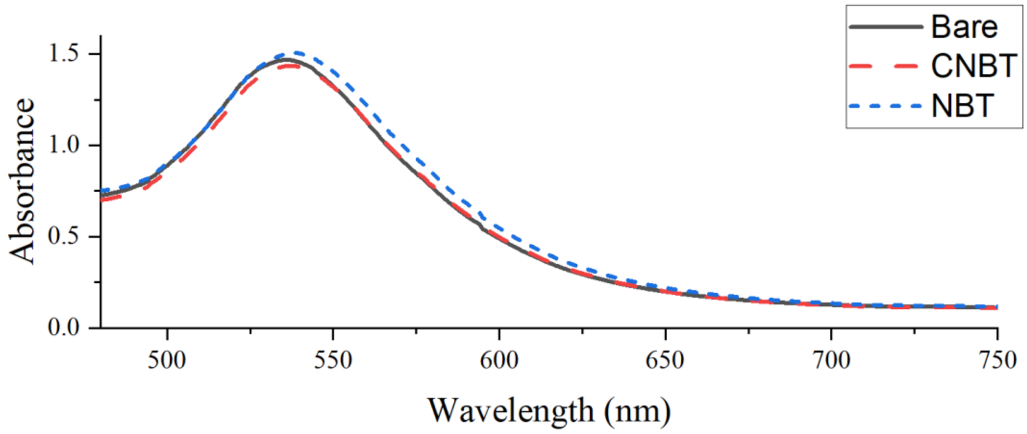

(**a**) UV-visible spectrum of unmodified (DSP linker only, black), DSP linker and CNBT (red) modified, and DSP linker and NBT (blue) modified showing the LSPR peak at 539 nm.

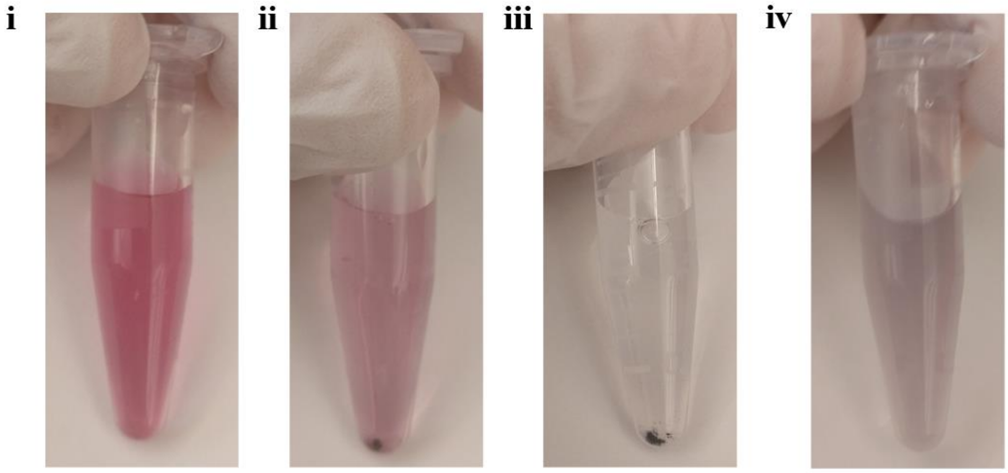

(**b**) Images of AuNPs treated with DSP linker and 4-nitrobenzenethiol (i), 4-methoxythiophenol (ii), and 4-aminothiophenol (iii) exhibiting no, partial, and total precipitation following centrifugation, respectively. AuNPs treated with 4-aminothiophenol prior to centrifugation (iv) exhibit rapid color change (spectral red-shift) associated with nanoparticle aggregation before precipitation occurs.

Figure S1. Surface Plasmon Resonance (LSPR) peak for unmodified and modified 60 nm gold nanoparticles (AuNPs).
